# Multimodality Screening For (Peri)Myocarditis In Newly Diagnosed Idiopathic Inflammatory Myopathies: A Cross-Sectional Study

**DOI:** 10.3233/JND-221582

**Published:** 2023-03-07

**Authors:** Johan Lim, Hannah A.W. Walter, Rianne A.C.M. de Bruin-Bon, Myrthe C. Jarings, R. Nils Planken, Wouter E.M. Kok, Joost Raaphorst, Yigal M. Pinto, Ahmad S. Amin, S. Matthijs Boekholdt, Anneke J. van der Kooi

**Affiliations:** a Department of Neurology, Amsterdam UMC, University of Amsterdam, Amsterdam Neuroscience, Amsterdam, The Netherlands; b Department of Cardiology, Amsterdam UMC, University of Amsterdam, Amsterdam, The Netherlands; c Department of Radiology and Nuclear Medicine, Amsterdam UMC, University of Amsterdam, Amsterdam Cardiovascular Science - Atherosclerosis & Ischemic Syndromes, Amsterdam, The Netherlands

**Keywords:** Neuromuscular diseases, muscular diseases, myositis, heart diseases

## Abstract

**Background:**

Cardiac involvement in idiopathic inflammatory myopathy (IIM or “myositis”) is associated with an approximate 4% mortality, but standardised screening strategies are lacking.

**Objective:**

We explored a multimodality screening on potentially reversible cardiac involvement –i.e. active (peri)myocarditis –in newly diagnosed IIM.

**Methods:**

We included adult IIM patients from 2017 to 2020. At time of diagnosis, patients underwent cardiac evaluation including laboratory biomarkers, electrocardiography, echocardiography, and cardiac magnetic resonance imaging (CMR). Based on 2019 consensus criteria for myocarditis, an adjudication committee made diagnoses of definite, probable, possible or no (peri)myocarditis. We explored diagnostic values of sequentially added diagnostic modalities by Constructing Classification and Regression Tree (CART) analysis in patients with definite/probable versus no (peri)myocarditis.

**Results:**

We included 34 IIM patients, in whom diagnoses of definite (six, 18%), probable (two, 6%), possible (11, 32%), or no (peri)myocarditis (15, 44%) were adjudicated. CART-analysis showed high-sensitivity cardiac troponin T (cut-off value < 2.3 times the upper limit of normal (xULN)) ruled out (peri)myocarditis with a sensitivity of 88%, while high-sensitivity troponin I (cut-off value > 2.9 xULN for females and > 1.8 xULN for males) ruled in (peri)myocarditis with a specificity of 100%. Applying high-sensitivity cardiac troponins with these cut-off values in a diagnostic algorithm without and with a CMR to the total population of 34 patients demonstrated a diagnostic accuracy for a clear diagnosis of probable/definite or no (peri)myocarditis of 59% and 68%, respectively.

**Conclusions:**

A diagnostic algorithm for detection of (peri)myocarditis in adult IIM may consist of sequential testing with high-sensitivity cardiac troponins and CMR.

## INTRODUCTION

Cardiac involvement in patients with idiopathic inflammatory myopathy (IIM; commonly referred to as “myositis”), may result in arrhythmias and/or cardiac failure with subsequent mortality in approximately 4% of patients, especially if left untreated [1]. A subset of patients with early stage cardiac involvement –i.e. active (peri)myocarditis –may be amenable to treatment [2].

The diagnosis of myocarditis (from various causes) has been based in the past on the Lake Louise criteria of 2009, targeting on tissue inflammation mainly with the help of conventional cardiac magnetic resonance imaging (CMR) techniques by assessment of late gadolinium enhancement (LGE) and by T2-weighted imaging [3]. In 2018, the criteria have been refined using novel CMR techniques for demonstrating myocardial oedema on T2-weighted imaging and native T2 mapping and for diffuse fibrosis and infiltrations using T1-imaging [4]. With these newer criteria, sensitivity for diagnosing acute myocarditis has increased from 74% to 85% by adding T1-mapping [5]. The latter criteria have also been adopted in a multimodality diagnostic strategy to detect immune-mediated myocarditis in cancer therapeutics, that translated probabilities in diagnostic categories of definite or probable versus possible and no myocarditis [6]. The Lake Louise criteria of 2009 and the newer CMR criteria of 2018 for myocarditis have both been validated with clinical criteria and endomyocardial biopsy [3–5]. Although it would suffice to perform CMR to diagnose acute myocarditis in IIM patients, a multimodality cardiac screening is often performed using laboratory testing, electrocardiography, echocardiography, and coronary angiography in addition to CMR, because of the predisposition of patients to other (cardio)vascular disease which may contribute to the CMR findings [7–9]. Diagnosing active (peri)myocarditis within a multimodality approach however has a priority for treatment with immune-suppressant agents and is the focus of the present study. We tested a multimodality screening strategy in newly diagnosed IIM patients with the hypothesis that it would 1) identify a considerable proportion of patients with active (peri)myocarditis and 2) make it possible to devise screening strategies for (peri)myocarditis. For these reasons, we designed this cross-sectional study.

## MATERIALS AND METHODS

### Patients

We included newly diagnosed adult IIM patients between February 2017 and February 2020 at a single referral centre for IIM in Amsterdam, The Netherlands. All patients were evaluated by an IIM specialist. Inclusion criteria were as follows: newly diagnosed adult patients with IIM based on the 2004 European Neuromuscular Centre (ENMC) criteria except inclusion body myositis [10], who were further classified into the following IIM subtypes: dermatomyositis [10–12], anti-synthetase syndrome [11], immune-mediated necrotising myopathy [10, 11, 13], polymyositis [10, 11], and non-specific/overlap myositis [10, 11]. This study was conducted in the setting of routine clinical practice and in accordance with the local research code provided by the IRB, national legislation, and the declaration of Helsinki. We registered the following baseline data at time of diagnosis: IIM subtype, age, sex, disease duration (time between first symptoms and diagnosis), muscular and extramuscular involvement/disease activity based on the Core Set Measures (CSMs) of the International Myositis Assessment & Clinical Studies (IMACS) Group (see Supplementary Material) [14], presence of an associated connective tissue disorder (CTD), presence of associated cancer, presence of myositis related autoantibodies as assessed by a line blot assay, presence of anti-mitochondrial autoantibodies (AMAs) as assessed by immunofluorescence, and prior or active immunosuppressive treatment.

### Cardiac screening

All patients were evaluated by a cardiologist with expertise in cardiomyopathies. We registered the following items at time of diagnosis: pre-existing cardiovascular disease (CVD), cardiovascular risk factors (CVRF; e.g. smoking, diabetes, hypertension), presence of symptoms of possible cardiac origin (i.e. complaints of dyspnoea, palpitations, peripheral oedema, chest pain, or syncope not attributable to another non-(peri)myocarditis diagnosis), and timing of cardiac evaluation.

At time of IIM diagnosis, patients underwent laboratory investigations, ECG, echocardiography, and CMR. Laboratory biomarkers included serum creatine kinase (CK) activity (reference value 145 IU/L for females and 171 IU/L for males), high-sensitivity cardiac troponin T (hs-TnT; reference value < 50 ng/L; Elecsys^®^ Troponin T high sensitive, Roche Diagnostics, Rotkreuz, Switzerland), high-sensitivity cardiac troponin I (hs-TnI; reference value females < 12 ng/L and males < 20 ng/L; high-sensitivity Troponin I, Beckman Coulter, Brea, California, USA), and N-terminal pro B-type natriuretic peptide (NT-proBNP; reference value < 130 ng/L; Elecsys^®^ proBNP II, Roche Diagnostics, Rotkreuz, Switzerland). Values were expressed as times the upper limit of normal.

A standard 12-lead ECG was recorded. Echocardiography was performed by a dedicated technician (R.B.). Echocardiographic views were acquired on a Vivid E9 or E95 (GE Healthcare, Horten, Norway) and were evaluated for wall motion abnormalities, systolic and diastolic ventricular function and pulmonary artery hypertension (PAH) according to guideline recommendations [15, 16]. Additional strain analyses –with strain abnormalities based on global longitudinal strain and mechanical dispersion –were performed offline on EchoPAC PC software v.201 (GE Healthcare, Horten, Norway) [17].

CMR images were acquired using 1.5-Tesla CMR (Magnetom Avanto, Siemens Medical Systems, Erlangen, Germany). CMR investigations consisted of steady-state free precession cine imaging in standard long axis images (2-chamber, 3-chamber, 4-chamber orientations) and a stack of short axis images. T2-weighted turbo spin echo or spectral attenuated inversion recovery sequences were acquired for detection of myocardial oedema. Post-contrast images were acquired for the detection of late gadolinium enhancement (LGE). Short axis cine imaging with full right ventricle and left ventricle coverage and a 20% inter-slice gap were segmented using dedicated post-processing software (Circle CV, Calgary, Canada). Papillary muscles were included in the left ventricular volume, not separately segmented. Additional parametric mapping consisting of T1/T2 mapping and calculation of extracellular volume (ECV) was performed in part of the patients [18, 19]. Parametric T1/T2 maps were obtained in 3 short axis views, and were assessed visually for regional differences. If these were observed, a ROI was used to quantify the regional T1/T2 values respectively. No average T1/T2 values across the entire myocardium in the short axis views were quantified, as this approach may mask regional abnormalities. Pending definitive local T1/T2 mapping reference values, < 1050 ms for T1-mapping and < 45 ms for T2-mapping were considered normal, while > 1100 ms for T1-mapping and > 50 ms for T2-mapping were considered abnormal. Additional coronary angiography to exclude coronary heart disease was performed at the discretion of the treating cardiologist.

An adjudication committee (J.L., H.B., A.A., Y.P., S.B, A.K.) assessed the presence of IIM-related (peri)myocarditis using consensus criteria for myocarditis, with designations of definite, probable, possible, or no (peri)myocarditis (6).

### Statistical analysis

Results were described using descriptive statistics. Sample size: convenience sample. Associations between the presence of probable/definite (peri)myocarditis and (extramuscular) disease activity measures were expressed as Spearman correlation coefficients (r_s_). In view of the explorative nature of this pilot study we did not correct for multiple comparison [20]. Diagnostic values of sequential diagnostic modalities (i.e. hs-TnT, hs-TnI, NT-proBNP, presence of AMAs, ECG abnormalities, echocardiography abnormalities, and CMR abnormalities) were explored by Constructing Classification and Regression Tree (CART) analysis using SPSS Statistics version 24.0 (IBM Corp., Armonk, NY, USA). CART analysis was first performed in a selection of patients with clear diagnoses of probable/definite or no (peri)myocarditis. Cut-off values of the troponins were determined as those with high specificity for the diagnosis (peri)myocarditis (rule in), and cut-off values with high sensitivity were used to rule out (peri)myocarditis. The most useful diagnostic modalities in CART were then ranked in order of appearance, to establish a diagnostic algorithm that was then tested for its accuracy in the total cohort of patients. Any data not published within the article –e.g. the CMR exam card/sequence protocol –will (after anonymization) be shared upon request from any qualified investigator.

## RESULTS

### Patient characteristics

We included 34 newly diagnosed IIM patients of whom the demographics and clinical features are summarised in Table 1. Detailed information on the ECG findings is available in the Supplementary Material. There were 10 patients (29%) who had received prior treatment with immunosuppressants, of whom five patients with partial but insufficient treatment response.

**Table 1:** Demographics and clinical features of the 34 included patients

	All (*n* = 34)	Probable/definite (peri)myocarditis (*n* = 8)	Possible (peri)myocarditis (*n* = 11)	No (peri)myocarditis (*n* = 15)
Demographics and disease features				
Disease subtype	- DM (*n* = 17)- ASS (*n* = 4)- IMNM (*n* = 7)- NM/OM (*n* = 6)	- DM (*n* = 2)- ASS (*n* = 1)- IMNM (*n* = 2)- NM/OM (*n* = 3)	- DM (*n* = 4)- ASS (*n* = 2)- IMNM (*n* = 4)- NM/OM (*n* = 1)	- DM (*n* = 11)- ASS (*n* = 1)- IMNM (*n* = 1)- NM/OM (*n* = 2)
Age (median years, IQR)	53 (42 to 62)	50 (34–59)	60 (47–66)	50 (37–62)
Females (n, %)	24 (71%)	5 (63%)	6 (55%)	13 (87%)
Disease duration (median months, IQR)	5 (3–10)	5 (3–10)	6 (2–8)	5 (3–13)
cardiovascular risk factors (n, %)	16 (47%)	4 (50%)	9 (82%)	3 (20%)
Prior cardiovascular disease (n, %)	7 (21%)	1 (13%)	5 (45%)	1 (7%)
Connective tissue disease (n, %)	4 (12%)	4 (50%)	0 (0%)	0 (0%)
Cancer (n, %)	1 (3%)	0 (0%)	0 (0%)	1 (7%)
Symptoms of possible cardiac origin (n, %)	23 (68%)	6 (75%)	10 (91%)	7 (47%)
Disease activity
Physician Global activity (median, IQR)	3.5 (2.5–4.1)	4.5 (4.0–6.0)	3.8 (1.9–4.4)	3.0 (2.3–3.5)
Patient Global activity (median, IQR)	6.1 (5.0–7.5)	7.5 (7.5–9.9)	5.9 (4.6–7.0)	5.9 (4.4–7.6)
MMT8 (median, IQR)	67 (59–72)	63 (54–64)	68 (59–73)	69 (63–73)
Health Assessment Questionnaire (median score 0–3, IQR)	1.9 (1.3–2.4)	2.1 (2.0–2.6)	1.9 (1.4–2.5)	1.5 (1.0–2.1)
Serum creatine kinase activity (median xULN, IQR)	- females: 15 (2.6–33) - males: 13 (2.2–28)	- females: 33 (3.7–65) - males: 28 (3.2–55)	- females: 21 (12–45) - males: 18 (10–38)	- females: 21 (12–45) - males: 6.3 (0.6–17)
Extramuscular disease activity	- VAS: 2.0 (1.2–2.6)	- VAS: 2.5 (1.5–3.3)	- VAS: 1.9 (1.2–2.3)	- VAS: 2.2 (1.0–2.8)
- VAS (median, IQR)	- any: 31 (91%)	- any: 7 (88%)	- any: 10 (91%)	- any: 14 (93%)
- extramuscular involvement (n, %)	- constitutional: 29 (85%) - cutaneous: 21 (62%) - skeletal: 9 (26%) - gastrointestinal: 0 (0%) - pulmonary: 4 (12%) - other: 16 (47%)	- constitutional: 8 (100%) - cutaneous: 8 (100%) - skeletal: 2 (25%) - gastrointestinal: 0 (0%) - pulmonary: 1 (13%) - other: 6 (75%)	- constitutional: 9 (82%) - cutaneous: 9 (82%) - skeletal: 2 (18%) - gastrointestinal: 0 (0%) - pulmonary: 1 (9%) - other: 3 (27%)	- constitutional: 12 (80%) - cutaneous: 4 (27%) - skeletal: 5 (33%) - gastrointestinal: 0 (0%) - pulmonary: 2 (13%) - other: 7 (47%)
Ancillary investigations
Autoantibodies	AMAs (*n* = 1)anti-EJ (*n* = 1)anti-HMGCR (*n* = 3)anti-Jo1 (*n* = 2)anti-Ku (*n* = 2)anti-MDA5 (*n* = 3)anti-Mi2 (*n* = 4)anti-NXP2 (*n* = 2)anti-PL7 (*n* = 1)anti-PMScl (*n* = 1)anti-Ro52 (*n* = 4)anti-SRP (*n* = 2)anti-TIF1γ (*n* = 2)seronegative (*n* = 9)	AMAs (*n* = 0)anti-EJ (*n* = 1)anti-Mi2 (*n* = 1)Anti-TIF1γ+ anti-Ku (*n* = 1)anti-Ro52 (*n* = 2)anti-SRP (*n* = 1)seronegative (*n* = 3)	AMAs (*n* = 0)anti-HMGCR (*n* = 2)anti-Jo1 (*n* = 1)anti-Ku (*n* = 1)anti-Mi2 (*n* = 1)anti-NXP2 (*n* = 1)anti-PL7 (*n* = 1)anti-SRP (*n* = 1)seronegative (*n* = 2)	AMAs (*n* = 1)anti-HMGCR (*n* = 1)anti-Jo1 (*n* = 1)anti-Ku (*n* = 1)anti-MDA5 (*n* = 3)anti-Mi2 (*n* = 2)anti-NXP2 (*n* = 1)anti-PMScl (*n* = 1)anti-Ro52 (*n* = 2)anti-TIF1γ (*n* = 1)seronegative (*n* = 4)
hs-TnT (median xULN, IQR)	3.1 (0.9–6.7)	11 (3.1–17)	3.2 (2.0–6.7)	1.0 (0.62–4.1)
hs-TnI (median xULN, IQR)	- females: 1.1 (0.3–2.9)- males: 0.7 (0.2–1.8)	- females: 3.2 (1.0–7.6)- males: 2.0 (0.6–4.6)	- females: 1.3 (0.4–6.4)- males: 0.8 (0.3–3.9)	- females: 0.6 (0.2–1.6)- males: 0.4 (0.1–1.0)
NT-proBNP (median xULN, IQR)	1.1 (0.6–2.2)	1.6 (0.7–3.2)	1.0 (0.6–4.2)	1.1 (0.6–1.4)
Abnormal ECG rest (n, %)	8 (24%)	4 (50%)	4 (36%)	0 (0%)
Abnormal ECG strain (n, %)	0 (0%)	0 (0%)	0 (0%)	0 (0%)
Abnormal echocardiography wall motion^a^ (n, %)	5 (15%)	2 (25%)	1 (9%)	2 (13%)
Abnormal echocardiography LVDD^a^ (n, %)	3 (9%)	0 (0%)	2 (18%)	1 (7%)
Abnormal echocardiography strain^a^ (n, %)	17 (50%)	5 (63%)	6 (55%)	6 (40%)
Abnormal CMR^b^ (n, %)	9 (26%)	8 (100%)	1 (100%)	0 (0%)

Abbreviations (alphabetical order): AMAs = anti-mitochondrial autoantibodies; ASS = anti-syntethase syndrom; CMR = cardiac magnetic resonance imaging; DM = dermatomyositis; ECG = electrocardiography; hs-TnI = high-sensitivity cardiac troponin I; hs-TnT = high-sensitivity cardiac troponin T; IMNM = immune-mediated necrotising myopathy; IQR = interquartile range; LVDD = left ventricular diastolic dysfuntion; MMT8 = manual muscle testing 8; NM/OM = non-specific/overlap myositis; NT-proBNP = N-terminal pro hormone B-type natriuretic peptid; xULN = times upper limit of normal. ^a^ Conventional echocardiography was assessed according to guideline recommendations [15, 16]. Strain echocardiography was considered abnormal based on global longitudinal strain (reference value –18 to–22%) and mechanical dispersion (reference value < 38 ms). ^b^ CMR consisted of conventional and in part of patients also parametric T1/T2 mapping. Pending definitive local T1/T2 mapping reference values, < 1050 ms for T1-mapping and < 45 ms for T2-mapping were considered normal, while > 1100 ms for T1-mapping and > 50 ms for T2-mapping were considered abnormal.

### Cardiac screening results

Seven patients (21%) had pre-existing CVD and 16 (47%) had one or more CVRFs at time of diagnosis. Twenty-three patients (68%) had one or more symptoms of possible cardiac origin, mostly consisting of dyspnoea (19 patients), to a lesser degree palpitations (nine patients) and chest pain (six patients), and no symptoms of syncope or peripheral oedema. Median time between start of treatment and ancillary investigations was as follows: zero days for laboratory biomarkers (IQR –3 to 0 days), one day for ECG (IQR –0.5 to 28 days), one day for echocardiography (IQR –2 to 12 days), and 23 days for CMR (IQR 2 to 48 days).

A diagnosis of definite (peri)myocarditis was adjudicated in six patients (18%), probable (peri)myocarditis in two patients (6%), possible (peri)myocarditis in 11 patients (32%), and no (peri)myocarditis in 15 patients (44%).The eight cases with diagnoses of probable/definite (peri)myocarditis were found in patients of all IIM subtypes (Table 2). Two out of these eight (25%) were without any cardiac symptoms, of whom one patient with progressive conduction abnormalities for which a pacemaker implantation was required (patient IMNM2Table 2). One patient with probable (peri)myocarditis had PAH (patient IMNM9 Table 2), none of the patients with a diagnosis of probable/definite (peri)myocarditis suffered from heart failure or had left ventricular diastolic dysfunction (LVDD; Table 2).

**Table 2:** Patient characteristics of 8 patients with a probable/definite diagnosis of (peri)myocarditis

Patient	Age	Sex	History	Cardiac symptoms	Lab biomarkers (xULN)	ECG	Echo- cardiography^a^	CMR^b^	Vascular imaging	(peri) myocarditis
DM10, anti-Mi2+	31	F	-	chest pain	- hs-TnT: 14 - hs-TnI: 5.3 - NTproBNP: 0.83	normal	- WMA: no - LVDD: no - strain: normal	- T2/LGE abnormalities: no - T1/T2M abnormalities: T1M (anteroseptal 1109 ms), ECV (31%), and T2M anteroseptal (61 ms)	NA	definite
DM15, anti-TIF1γ+, anti-Ku+	60	M	-	dyspnea, palpitations	- hs-TnT: 2.3 - hs-TnI: 0.46 - NTproBNP: 0.45	normal	NA	- T2/LGE abnormalities: suggestive LGE inferobasal-subendocardial - T1/T2M abnormalities: T1M inferobasal (1165 ms) and T2M inferobasal (58 ms)	CAG: normal	definite
ASS6, anti-EJ+, anti-Ro52+	46	F	-	chest pain, dyspnea	- hs-TnT: 7.0 - hs-TnI: 1.6 - NTproBNP: 0.64	normal	- WMA: yes - LVDD: no - strain: abnormal GLS (-14%) and MD (50 ms)	- T2/LGE abnormalities: no - T1/T2M abnormalities: T1M diffuse (1141 ms), ECV (33%), T2M septal (57 ms)	NA	definite
IMNM2, anti-Ro52+	56	M	smoking, SSc	-	- hs-TnT: 29 - hs-TnI: 38 - NTproBNP: 5.8	abnormal conduction requiring pacemaker	- WMA: no - LVDD: no - strain: abnormal MD (40 ms)	- T2/LGE abnormalities: LGE anterolateral-subendocardial - T1/T2M abnormalities: T1M subendocardial (1158 ms) and ECV (37%)	CAG: normal	definite
IMNM9, anti-SRP+	53	M	smoking, OSAS	-	- hs-TnT: 17 - hs-TnI: 5.0 - NTproBNP: 1,4	aspecific VPBs^c^	- WMA: no - LVDD: no - strain: abnormal GLS (-17.5%) and MD (59 ms)	- T2/LGE abnormalities: suggestive LGE basolateral-epi/midmyocardial - T1/T2M abnormalities: T1M inferobasoseptal (1070 ms) and ECV (37%)	chest CT: dilated pulmonary trunk of 3.8 cm	probable
NM/OM1, seronegative	72	F	MCTD	palpitations	- hs-TnT: 5.5 - hs-TnI: 3.5 - NTproBNP: 3.5	abnormal Q waves consistent with CHD^c^	- WMA: no - LVDD: no - strain: abnormal GLS (-14%) and MD (49 ms)	- T2/LGE abnormalities: T2 hyperintensity and suggestive LGE inferobasolateral-epicardial - T1/T2M abnormalities: T1M inferobasolateral (1160 ms)	NA	definite
NM/OM4, Seronegative	25	F	MCTD	chest pain, dyspnoea, palpitations	- hs-TnT: 17 - hs-TnI: 3.0 - NTproBNP: 1.7	abnormal T waves	- WMA: yes - LVDD: no - strain: abnormal GLS (-17%) and MD (43 ms) - minimal PE	- T2/LGE abnormalities: LGE basal pericardial - T1/T2M abnormalities: no	NA	definite
NM/OM6, Seronegative	41	F	-	dyspnea	- hs-TnT: 0.2 - hs-TnI: 0.1 - NTproBNP: 2.1	normal	- WMA: no - LVDD: no - strain: normal	- T2/LGE abnormalities: no - T1/T2M abnormalities: suggestive T2M septal (55 ms)	NA	probable

Abbreviations (alphabetical order): CMR = cardiac magnetic resonance imaging; ECG = electrocardiography; ECV = extracellular volume; *F* = female, GLS = global longitudinal strain (reference value –18 to–22%); hs-TnI = high-sensitivity cardiac troponin I; hs-TnT = high-sensitivity cardiac troponin T; LGE = late gadolinium enhancement; LVDD = left ventricular diastolic dysfunction; *M* = male; MD = mechanical dispersion (reference value < 38 ms); NA = not available; NT-proBNP = N-terminal pro hormone B-type natriuretic peptid; PE = pericardial effusion; T1M = native T1-mapping; T2 = T2-weighted imaging; T2M = native T2 mapping. ^a^ Conventional and strain echocardiography were assessed and when considered abnormal, absolute strain values are shown in parentheses. ^b^ CMR consisted of conventional and in part of patients also parametric T1/T2 mapping and when considered abnormal, absolute values for parametric T1/T2 mapping are shown in parentheses. Pending definitive local T1/T2 mapping reference values, < 1050 ms for T1-mapping and < 45 ms for T2-mapping were considered normal, while > 1100 ms for T1-mapping and > 50 ms for T2-mapping were considered abnormal. ^c^ Abnormalities were not considered disease-related by the adjudication committee (J.L., H.B., A.A., Y.P., S.B, A.K.).

Eleven patients (32%) had a diagnosis of possible (peri)myocarditis based on abnormal hs-TnT and/or hs-TnI levels with either symptoms or ECG abnormalities of possible (peri)myocarditis origin. CMR was normal in all but one patient with long-standing CMR abnormalities suggestive of (peri)myocarditis in whom other causes of cardiomyopathy –e.g. genetic –could not be completely ruled out. Of the 15 patients without a diagnosis of (peri)myocarditis, seven patients (47%) had abnormal levels of hs-TnT and five patients (33%) had abnormal levels of hs-TnI.

### Association between (peri)myocarditis and (extramuscular) disease activity

We had complete data on muscle and extramuscular disease activity available for 30/34 patients (88%); missing data were excluded pairwise for calculating the Spearman correlation coefficient. We found significant associations with a moderate effect size between the presence of probable/definite (peri)myocarditis and the following disease activity measures: Physician Global Activity Visual Analog Scale (VAS) (correlation coefficient 0.47, *p* = < 0.01; 2-sided Spearman correlation coefficient), Patient Global Activity VAS (correlation coefficient 0.47, *p* = < 0.01; 2-sided Spearman correlation coefficient), Health Assessment Questionnaire (correlation coefficient 0.44, *p* = 0.02; 2-sided Spearman correlation coefficient), and MMT8 (correlation coefficient 0.43, *p* = 0.02; 2-sided Spearman correlation coefficient). We found no significant associations between the presence of probable/definite (peri)myocarditis and serum CK activity (correlation coefficient 0.19, *p* = 0.28; 2-sided Spearman correlation coefficient) or extramuscular disease activity VAS (correlation coefficient 0.19, *p* = 0.32; 2-sided Spearman correlation coefficient).

### Screening strategies for (peri)myocarditis

CART analysis confirmed that CMR was the diagnostic modality identifying all patients with a clear diagnosis of probable/definite (peri)myocarditis or no (peri)myocarditis (Supplementary Material). In contrast, neither abnormally elevated NT-proBNP, the presence of AMAs, abnormalities on ECG, nor abnormalities on echocardiography appeared to have added value in discriminating between patients with a diagnosis of probable/definite (peri)myocarditis and patients with no evidence of (peri)myocarditis (Supplementary Material). Furthermore, CART analysis showed that both high-sensitivity cardiac troponins were useful as in a diagnostic algorithm, with hs-TnT most useful to rule-out (peri)myocarditis and hs-TnI most useful to rule-in (peri)myocarditis (Supplementary Material). A hs-TnT with a cut-off value of < 2.3 xULN was deemed optimal to rule-out (peri)myocarditis with sensitivity of 88%, specificity of 67%, and negative predictive value of 91%. hs-TnI with a cut-off value of > 2.9 xULN for females and > 1.8 xULN for males was selected as optimal to rule-in (peri)myocarditis with sensitivity of 63%, specificity of 100% and positive predictive value of 100%. Combining hs-TnT to rule-out (peri)myocarditis and hs-TnI to rule-in (peri)myocarditis in a diagnostic algorithm showed 87% diagnostic accuracy for a correct diagnosis (Supplementary Material). When applied to the total cohort of 34 patients (including patients with a diagnosis of possible (peri)myocarditis), diagnostic accuracy was 59%, and when combined with CMR as second step, diagnostic accuracy was 68% (Fig. 3). Hence, we propose the following diagnostic algorithm that combines hs-TnT, hs-TnI, and CMR (Fig. 4).

**Fig. 1:**
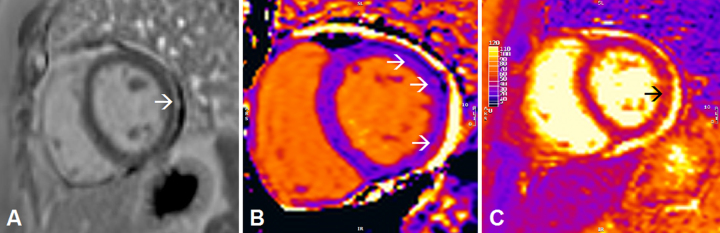
Abnormalities on cardiac magnetic resonance imaging (CMR) in short-axis view consistent with (peri)myocarditis in a patient with non-specific myositis/overlap myositis. Classic CMR (peri)myocarditis abnormalities: late gadolinium enhancement indicating myocardial fibrosis is seen epicardial in the basal-mid inferolateraal segment on T1-weighted imaging. Parametric T1/T2-mapping allows for quantitative (re-)evaluation of (peri)myocarditis abnormalities: increased values on T1-mapping (arrows Fig. 1B) and T2-mapping (arrow Fig. 1C) indicating myocardial oedema are (focally) seen in the inferolateral segment (short axis view).

**Fig. 3:**
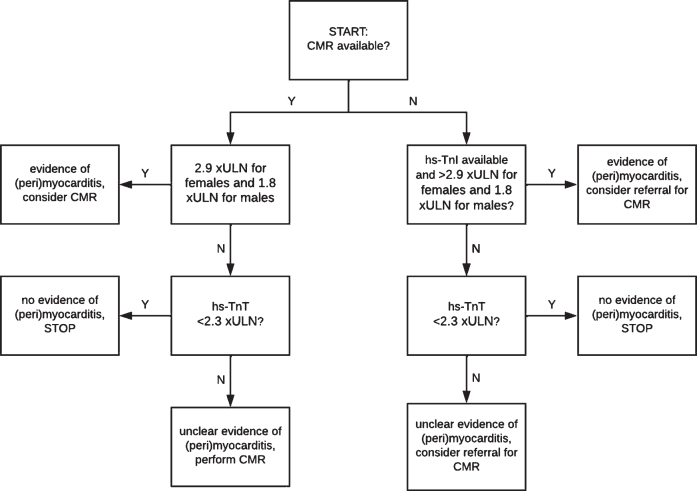
Proposed diagnostic algorithm as screening for (peri)myocarditis. Note that this diagnostic algorithm is open for improvement and validation and is not a final algorithm for clinical use. Abbreviations (alphabetical order): CMR = cardiac magnetic resonance imaging; hs-TnI = high-sensitivity cardiac troponin; hs-TnT = high-sensitivity cardiac troponin T; xULN = times the upper limit of normal.

## DISCUSSION

Our study confirms the findings of previous reports that (peri)myocarditis/early myocardial involvement is found in a considerable proportion (almost one fifth) of newly diagnosed IIM patients [2, 7–9, 21–23]. Our study adds to the literature that a multimodality screening strategy for the purpose of diagnosing active (peri)myocarditis in IIM may be modified towards a strategy using two initial major diagnostic tools: a laboratory test (including both hs-TnT and hs-TnI) in combination with a comprehensive CMR investigation (that includes parametric T1/T2 mapping). Compared to two histopathological autopsy studies demonstrating active myocarditis in 25 to 30% of IIM patients [24, 25], we describe a somewhat lower frequency of active (peri)myocarditis in 18% of patients. Our lower frequency may be a result of selection of less severe patients than those of the autopsy reports, but may to some extent also be a result of an underestimation by restricting the diagnosis of (peri)myocarditis to those with a clear diagnosis and diagnostic delay of CMR in part of the patients. Our results may also be compared to a single study using CMR that demonstrated myocarditis in 75% of 20 acute IIM patients, following the Lake Louise CMR criteria for myocarditis of 2009 [7] Although some overestimation of true prevalence of active myocarditis is expected using the older criteria, the difference in false positive rates between older and the newer criteria is not larger than 3% and cannot fully explain the difference in prevalence between the studies [5]. Therefore we believe our results present a conservative estimate of the true prevalence of active myocarditis in IIM. Still other studies found cardiac abnormalities in up to 65% of IIM patients but their results were not restricted to (peri)myocarditis [9, 21, 23].

We found that a diagnosis of probable/definite (peri)myocarditis was significantly associated with global disease activity as assessed by both patients and physicians, muscle weakness as assessed by manual muscle testing, disability, but not with serum CK activity or extramuscular disease activity. It has been reported that serum CK activity lacks diagnostic accuracy [26–29], and this may in part explain the lack of correlation between diagnosis of probable/definite (peri)myocarditis and serum CK activity. This finding would appear however appear to conflict other studies that did find associations between cardiac involvement and serum CK activity [2, 23]. An updated systematic review and (if feasible) meta-analysis or a larger prospective cohort study may provide additional insights in associations between (peri)myocarditis/myocardial involvement on the one hand, and muscular and extramuscular involvement/disease activity on the other hand.

**Fig. 2:**
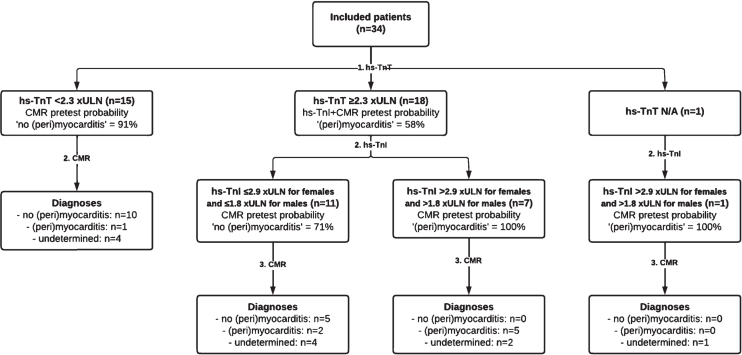
Diagnostic accuracy of combining high-sensitivity cardiac troponins T and I as first step, and cardiac magnetic resonance imaging as second step in the total population of 34 included patients. This resulted in a diagnostic accuracy of 68% (23 of 34 patients), leaving diagnosis undetermined in 11 patients (32%) despite performing a CMR. Abbreviations (alphabetical order): CMR = cardiac magnetic resonance imaging; hs-TnI = high-sensitivity cardiac troponin; hs-TnT = high-sensitivity cardiac troponin T; N/A = not available.

The importance of CMR in a standardised screening strategy that includes parametric T1/T2-mapping is illustrated by the detection of (peri)myocarditis in approximately a quarter to a third of IIM patients in whom (peri)myocarditis was not suspected based on symptoms of possible cardiac origin or conventional ancillary investigations –i.e. ECG, cardiac echography, and LGE on CMR - alone. As expected from the criteria in the definition that are used for the diagnosis of myocarditis that includes CMR findings [6]. CMR was the most useful diagnostic modality (Fig. 2) [8]. Similarly, our finding that approximately a third of patients with a diagnosis of probable/definite (peri)myocarditis had abnormalities on parametric T1/T2-mapping in CMR, but not with conventional LGE on CMR, is in accordance with earlier reports [2, 30]. Still, in 11 of 34 (32%) of our patients who had a CMR, diagnosis was classified as possible (peri)myocarditis, underlining the need for additional clues to confirm/exclude the diagnosis [6, 8, 31].

The limitations of CMR –that include availability, quality and costs –call for a gatekeeper to guide additional diagnostic workup or for a diagnostic equivalent for diagnosing (peri)myocarditis. The CART-analyses in our study suggest that both hs-TnT and hs-TnI have potential as gatekeepers to rule-out (peri)myocarditis and to rule-in (peri)myocarditis, respectively (Fig. 2). Our results –suggesting high sensitivity of hs-TnT and high specificity of hs-TnI –are in accordance with an earlier report that used normal reference ranges of second generation cardiac troponins as cut-off levels [32]. As of yet, there are no 100% accurate cut-off values for cardiac troponins in IIM [33]. Our CART analysis suggested a cut-off value of hs-TnT < 2.3 xULN for rule-out with a sensitivity of 88% and negative predictive value of 94%. When further validated, this cut-off level of hs-TnT may considerably reduce the number of CMRs needed (in our study reducing the number of CMRs from 42 to 23, an absolute reduction of 45%). Although CART analysis further suggested that hs-TnI with cut-off value of > 2.9 xULN for females and > 1.8 xULN for males may identify 63% of patients with probable/definite (peri)myocarditis with reasonable certainty (positive predictive value = 63%), there remains a need to confirm the diagnosis with CMR. There also remains a need to better delineate patients with intermediate levels of hs-TnT and hs-TnI and one also has to consider the added functional and structural information that CMR may provide.

Of note, it is unclear whether the “false positive” rates of hs-TnT found in our study and that of earlier reports reflect only “true” false positives, i.e. cross-reactivity of skeletal muscle troponin. It may also be that “false positive” hs-TnT and hs-TnI levels reflect subclinical (peri)myocarditis at least for a proportion of patients, in whom (peri)myocarditis is not (yet) visible even on state of the art CMR [34]. Other abnormalities, such as abnormally elevated levels of NT-proBNP, or the presence of AMAs, abnormalities on ECG, and abnormalities on echocardiography were not valuable in our diagnostic flowchart for a positive diagnosis of (peri)myocarditis in patients with newly diagnosed IIM (Fig. 2). Our findings appear to be in conflict with reports that suggested NT-proBNP and AMAs for discriminating between patients with and without a diagnosis of (peri)myocarditis [23, 35]. Differences in the applied criteria for defining (peri)myocarditis/myocardial involvement, the techniques of the ancillary investigations performed, and the studied populations may explain some of the differences in diagnostic accuracy.

Furthermore, while we did not find patients with cardiac failure at time of diagnosis, we did find LVDD in approximately one tenth of patients –none of whom had a diagnosis of probable/definite (peri)myocarditis –and PAH in one patient with a probable diagnosis of (peri)myocarditis. It is hypothesised that both LVDD and PAH are precursors of cardiac failure in IIM patients, leading to subsequent morbidity and mortality [23, 36, 37]. While the prevalence of LVDD and PAH in our study was in line with two earlier studies of IIM patients with Western-Northern European ethnicity [38, 39], it appeared lower than the prevalence [42–77% ] found in studies of IIM patients with East-Asian/Han-Chinese ethnicity [35, 40–42]. While differences in diagnostic criteria and definitions of LVDD may explain some of the discrepancies [35, 40–42], it is possible that diastolic abnormalities suggest subclinical (peri)myocarditis in IIM patients with East-Asian/Han-Chinese ethnicity in particular.

The strengths of our study are the multimodality assessment of (peri)myocarditis, including novel diagnostic modalities, the use of the most recent consensus criteria for (peri)myocarditis, and the use of the IMACS CSMs [6]. The main limitations of our study are the following: 1) the relative small sample size; 2) prior treatment with immunosuppressants and diagnostic delay/missing data regarding CMR investigations in some of the patients, that may have led to underestimating the number of patients with a diagnosis of probable/definite (peri)myocarditis; and [3] the use of CMR and additional criteria for (peri)myocarditis instead of results from myocardial biopsies as the basis of the adjudication committee’s assessment [6]. We used the criteria that were in accordance with consensus papers on diagnosis of myocarditis [4, 6], but were devised for cancer therapies-associated myocarditis. We however favoured their use as they provided a clinically useful division into probabilities of the diagnosis. Finally, other diagnostic modalities were not studied in our study that may have had added value (e.g. technetium pyrophosphate scintigraphy and positron emission tomography) [6, 31].

In conclusion, we show that routine multimodality screening for (peri)myocarditis in IIM at the time of diagnosis of IIM yields a considerable number of diagnoses of probable/definite (peri)myocarditis. A standardised screening strategy may however be more limited and consist of sequential testing with high-sensitivity cardiac troponins followed by CMR. As diagnostic uncertainty on the presence of (peri)myocarditis remained despite CMR and additional investigations in about a third of patients, our results are open for improvement and validation and are not a final algorithm for clinical use.

## Supplementary Material

Supplementary Material
