# Supplementary material for: Multimodality Screening For (Peri)Myocarditis In Newly Diagnosed Idiopathic Inflammatory Myopathies: A Cross-Sectional Study
Source: J Neuromuscul Dis. 2023 Mar 7;10(2):185–97. doi: 10.3233/JND-221582 (PMC10041435; doi:10.3233/JND-221582)
Supplement: Supplementary Material [file jnd-10-jnd221582-s001.docx]

**Supplementary Material**

Categories of Myocarditis^1^

*Definite Myocarditis*

Any of the following:

1. Tissue pathology diagnostic of myocarditis (e.g., on biopsy or autopsy)
2. Cardiac magnetic resonance imaging (CMR) diagnostic of myocarditis, a clinical syndrome and one of following:
   1. Elevated biomarker of cardiac myonecrosis
   2. Electrocardiography (ECG) evidence of (peri)myocarditis
3. New wall motion abnormality on echocardiogram not explained by another diagnosis (e.g., acute coronary syndrome, stress induced cardiomyopathy, sepsis) and all of the following:
   1. Clinical syndrome consistent with myocarditis
   2. Elevated biomarker of cardiac myonecrosis
   3. ECG evidence of (peri)myocarditis
   4. Negative angiography or other testing to
   5. Exclude obstructive coronary disease

*Probable Myocarditis*

Any of the scenarios below that are not explained by another diagnosis (eg, acute coronary syndrome, trauma, stress induced cardiomyopathy):

1. CMR with findings diagnostic of myocarditis without any of the following:
   1. Clinical syndrome consistent with myocarditis
   2. Elevated biomarker of cardiac myonecrosis
   3. ECG evidence of (peri)myocarditis
2. Nonspecific CMR findings suggestive of myocarditis with any one or more of the following:
   1. Clinical syndrome consistent with myocarditis
   2. Elevated biomarker of cardiac myonecrosis
   3. ECG evidence of (peri)myocarditis
3. New wall motion abnormality on echocardiogram with a clinical syndrome consistent with myocarditis and either:
   1. Elevated biomarker of cardiac myonecrosis
   2. ECG evidence of (peri)myocarditis
4. A scenario meeting criteria for Possible Myocarditis with18 fluorodeoxyglucose positron emission tomography imaging showing patchy cardiac fluorodeoxyglucose uptake without another explanation

*Possible myocarditis*

Any of the scenarios below that are not explained by another diagnosis (e.g., acute coronary syndrome, trauma, stress induced cardiomyopathy):

1. Nonspecific CMR findings suggestive of myocarditis with none of the following:
   1. Clinical syndrome consistent with myocarditis
   2. Elevated biomarker of cardiac myonecrosis
   3. ECG evidence of (peri)myocarditis
2. New wall motion abnormality on echocardiogram and one of the following:
   1. Clinical syndrome consistent with myocarditis
   2. ECG evidence of (peri)myocarditis
3. New elevated biomarker (beyond baseline) and one of the following:
   1. Clinical syndrome consistent with myocarditis
   2. ECG evidence of (peri)myocarditis

Muscular and extramuscular involvement/disease activity based on the Core Set Measures of the International Myositis Assessment & Clinical Studies Group

1. Physician Global Activity: assessment of global disease activity on a 10 cm Visual Analogue Scale by the treating physician
2. Patient Global Activity: assessment of global disease activity on a 10 cm Visual Analogue Scale by the patient
3. Manual Muscle Testing: sum score of 12 bilaterally scored muscle groups and two axial muscle groups.
   1. Bilaterally scored muscle groups: shoulder elevators, shoulder abductors, elbow flexors, wrist flexors, wrist extensors, hip flexors, hip extensors, hip abductors, knee flexors, knee extensors, ankle flexors, and ankle extensors. Axial muscle groups: neck flexors and neck extensors.
   2. Muscle strength assessment according to Kendall

0 = no contractions felt in the muscle

1 = moves through partial range of motion in horizontal plane

2 = moves through complete range of motion in horizontal plane

3 = moves through partial range of motion in antigravity position

4 = gradual release from test position in antigravity position

5 = holds test position (no added pressure) in antigravity position

6 = holds test position against slight pressure in antigravity position

7 = holds test position against slight to moderate pressure in antigravity position

8 = holds test position against moderate pressure in antigravity position

9 = holds test position against moderate to strong pressure in antigravity position

10 = holds test position against strong pressure in antigravity position

1. Health Assessment Questionnaire: the average of a questionnaire scoring eight domains from 0-3.
2. Domains: dressing and grooming, arising, eating, walking, hygiene, reach, grip, activities
3. Score

0 = without any difficulty

1 = with some difficulty

2 = with much difficulty

3 = unable to do

1. Serum muscle enzyme activities expressed as the most abnormal one in times the upper limit of normal of the following muscle-associated enzymes:
2. Creatine kinase
3. Alanine transferase
4. Aspartate aminotransferase
5. Lactate dehydrogenase
6. Extramuscular disease activity on a 10 cm Visual Analogue Scale based on the Myositis Disease Activity Assessment Tool in which, amongst others, the following domains were assessed
7. Constitutional disease activity: pyrexia, weight loss, fatigue/malaise/lethargy
8. Cutaneous disease activity: cutaneous ulceration, erythroderma, panniculitis, erythematous rashes, heliotrope rash, Gottron’s papules/sign, periungual capillary changes, alopecia, mechanic hands
9. Skeletal disease activity: arthritis, arthralgia
10. Gastrointestinal disease activity: dysphagia, abdominal pain related to the myositis disease process
11. Pulmonary disease activity: respiratory muscle weakness without interstitial lung disease (ILD), active reversible ILD, dysphonia
12. Cardiovascular disease activity: pericarditis, myocarditis, arrhythmia, sinus tachycardia
13. Other disease activity: e.g. subcutaneous edema, Raynaud phenomenon

ECG findings in the 34 included patients.

| DM1 | Normal | Normal |
| --- | --- | --- |
| DM2 | Normal | Normal |
| DM3 | Minor ST depression in II | Normal |
| DM4 | Normal | Normal |
| DM5 | Normal | n/a |
| DM6 | Normal | Normal |
| DM7 | Normal | n/a |
| DM8 | Normal | n/a |
| DM9 | Normal | n/a |
| DM10 | Normal | Normal |
| DM11 | Normal | n/a |
| DM12 | Normal | n/a |
| DM13 | Normal | n/a |
| DM14 | n/a | n/a |
| DM15 | Normal | n/a |
| DM16 | Normal | Normal |
| DM17 | Normal | n/a |
| IMNM1 | n/a | n/a |
| IMNM2 | Several PACs, left heart axis, anticus block and complete RBBB with secondary repolarisation abnormalities, ST depression in V5-6 | Normal |
| IMNM3 | Normal | Normal |
| IMNM7 | Possible left atrium hypertrophy, new anterior T wave abnormalities consistent with anterior wall infarction | Normal |
| IMNM8 | 1st degree AV block. Pathological Q waves in III and aVF consistent with inferior wall infarction. | n/a |
| IMNM9 | Double PACs, 1 PAC run with maximal frequency of 133 bpm and duration of 1 sec, 0.05% ectopic heart beats as percentage of total heart beats. Solitary and multiform PVCs in bi- and trigemini at a maximum of 504/hour and at an average of 201/hour, 4,5% ectopic heart beats as percentage of total heart beats. | Incomplete RBBB, PACs |
| IMNM10 | Normal | n/a |
| NM/OM1 | Pathological Q waves in V1-V2 consistent with inferior wall infarction | Normal |
| NM/OM3 | Normal | n/a |
| NM/OM4 | ST abnormalities consistent with pericarditis | n/a |
| NM/OM5 | borderline abnormal ECG with intraventricular conduction abnormality | n/a |
| NM/OM6 | Normal | n/a |
| NM/OM7 | Normal | n/a |
| ASS3 | Normal | n/a |
| ASS4 | Normal | n/a |
| ASS5 | Normal | n/a |
| ASS6 | Normal | Normal |

Screening strategies for (peri)myocarditis

First, we performed CART analysis combining all the diagnostic modalities/variables of interest, i.e. hs-TnT, hs-TnI, NT-proBNP, presence of AMAs, ECG abnormalities, echocardiography abnormalities, and CMR abnormalities. We found that CMR was the diagnostic modality identifying all patients with a clear diagnosis of probable/definite (peri)myocarditis or no (peri)myocarditis (Figure 1A).

Second, we performed CART analysis with hs-TnT forced as first variable and found that hs-TnT with a cut-off value of <5.3 xULN as a first step would have resulted in two false-negative diagnoses of probable/definite (peri)myocarditis (Figure 1B; patients DM15 and NM/OM6 Manuscript Table 2). One of these patients had a diagnosis of probable (peri)myocarditis, normal hs-TnT, and mild symptoms of possible cardiac origin in whom standard treatment with high-dose glucocorticoids resulted in improvement of these symptoms and resolution of CMR abnormalities (patient NM/OM6 Manuscript Table 2). The other patient had a diagnosis of definite (peri)myocarditis, hs-TnT of 2.3 xULN, and clinically relevant disease based on CMR abnormalities in the absence of an alternative ischemic cause (patient DM15 Manuscript Table 2). We optimised the cut-off value by manually choosing <2.3 xULN, resulting in one false-negative diagnosis/mild case of probable (peri)myocarditis (patient NM/OM6 Manuscript Table 2). Using this manually chosen cut-off value of <2.3 xULN, subsequent CART analysis showed that this was more optimal to rule-out (peri)myocarditis with sensitivity of 88%, specificity of 67%, and negative predictive value of 91% (Figure 1C).

Third, we performed CART analysis with hs-TnI forced as first variable and found that hs-TnI with a cut-off value of > 2.9 xULN for females and 1.8 xULN for males as a first step resulted in five patients diagnosed as (peri)myocarditis and zero false-positive diagnoses (Figure 1D). There were three false-negative diagnoses: apart from the two patients described above as having a hs-TnT of < 5.3 xULN, one additional patient with low hs-TnI had a clinically relevant (peri)myocarditis, based on CMR results and symptoms of possible cardiac origin (patient ASS6 Manuscript Table 2). Nevertheless, hs-TnI with a cut-off value of > 2.9 xULN for females and 1.8 xULN for males was selected as optimal to rule-in (peri)myocarditis with sensitivity of 63%, specificity of 100% and positive predictive value of 100%.

Fourth, we performed CART analysis with hs-TnT and hs-TnI and the abovementioned optimal cut-off levels in the 23 patients selected with a clear diagnosis of probable/definite or no (peri)myocarditis (Figure 1E). All patients with hs-TnT of <2.3 xULN also had hs-TnI of ≤ 2.9 xULN for females and 1.8 xULN for males, while patients with hs-TnT of ≥2.3 xULN could be divided in those with hs-TnI of ≤ 2.9 xULN for females and 1.8 xULN for males and > 2.9 xULN for females and 1.8 xULN for males. This resulted in a combined diagnostic accuracy for hs-TnT and hs-TnI of 87% (20 of 23 patients) in those with a clear diagnosis (Figure 1E).

When this combined diagnostic algorithm of hs-TnT and hs-TnI was applied in the total cohort of 34 patients (that included patients with a diagnosis of possible (peri)myocarditis), diagnostic accuracy decreased to 59% (20 of 34 patients). When this combined diagnostic algorithm of hs-TnT and hs-TnI was combined with CMR as second step in the total cohort of 34 patients (that included patients with a diagnosis of possible (peri)myocarditis), diagnostic accuracy increased to 68% (23 of 34 patients; Figure 3).


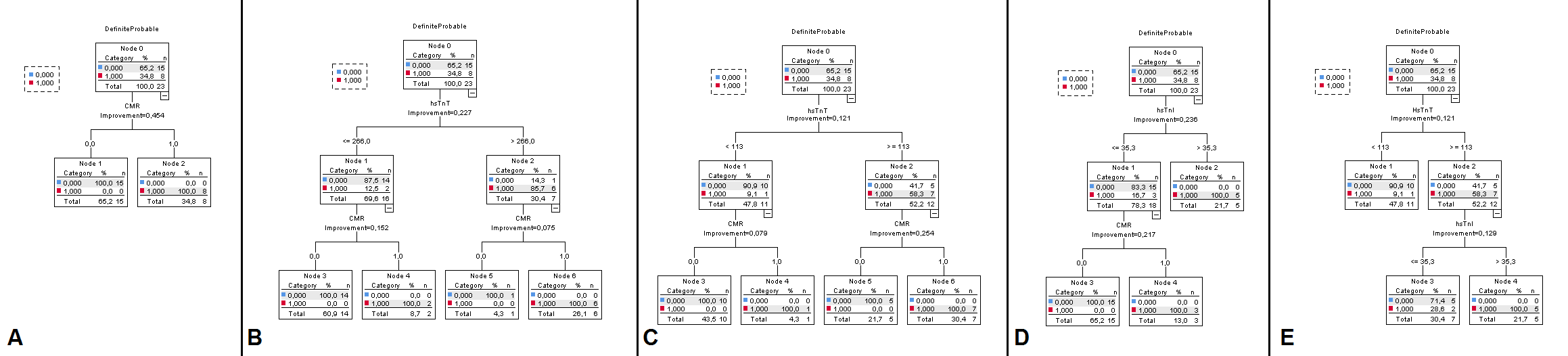


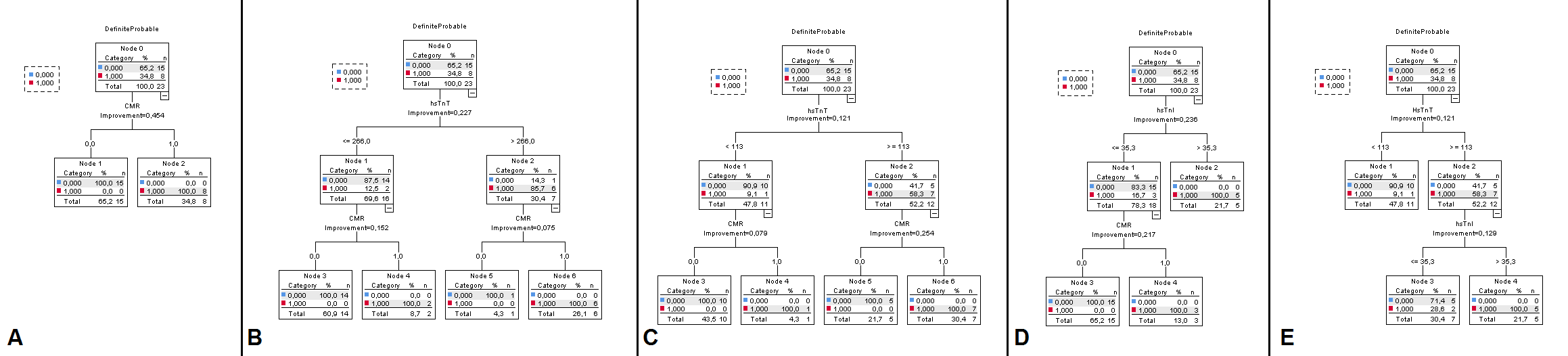


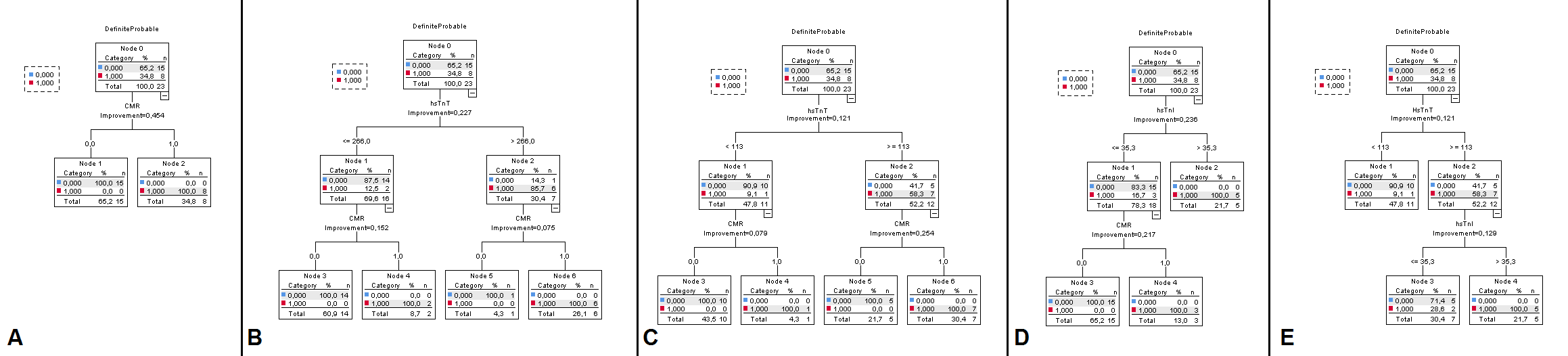


Figure 1. Diagnostic values of sequential diagnostic modalities were explored by Constructing Classification and Regression Tree (CART) analysis in patients with a diagnosis probable/definite (peri)myocarditis and patients with a diagnosis of no (peri)myocarditis. Cardiac magnetic resonance imaging (CMR) was the most useful diagnostic modality (Figure 2A), while high-sensitivity cardiac troponin T (hs-TnT) and high-sensitivity cardiac troponin I (hs-TnI) were useful as gatekeepers for CMR, using (manually chosen) <113 ng/L (= <2.3 times the upper limit of normal (x ULN)) to rule-out and >35 ng/L to rule-in (peri)myocarditis, respectively (Figure 1B-D). Twenty of 23 patients had a clearly predicted outcome combining hs-TnT to rule-out (peri)myocarditis and hs-TnI to rule-in (peri)myocarditis, resulting in a combined diagnostic accuracy of 87% (Figure 2E). Note: hs-TnT and hd-TnI values are expressed in absolute values for the analysis. Abbreviations (alphabetical order): CMR = cardiac magnetic resonance imaging; hs-TnI = high-sensitivity cardiac troponin; hs-TnT = high-sensitivity cardiac troponin .
